# Corticosteroid Treatment Ameliorates Acute Lung Injury Induced by 2009 Swine Origin Influenza A (H1N1) Virus in Mice

**DOI:** 10.1371/journal.pone.0044110

**Published:** 2012-08-29

**Authors:** Chenggang Li, Penghui Yang, Yanli Zhang, Yang Sun, Wei Wang, Zhen Zou, Li Xing, Zhongwei Chen, Chong Tang, Feng Guo, Jiejie Deng, Yan Zhao, Yiwu Yan, Jun Tang, Xiliang Wang, Chengyu Jiang

**Affiliations:** 1 State Key Laboratory of Medical Molecular Biology, Institute of Basic Medical Sciences, Chinese Academy of Medical Sciences, Department of Biochemistry and Molecular Biology, Peking Union Medical College; Tsinghua University, Beijing, China; 2 State Key Laboratory of Pathogen and Biosecurity, Beijing Institute of Microbiology and Epidemiology, Beijing, China; National University of Singapore, Singapore

## Abstract

**Background:**

The 2009 influenza pandemic affected people in almost all countries in the world, especially in younger age groups. During this time, the debate over whether to use corticosteroid treatment in severe influenza H1N1 infections patients resurfaced and was disputed by clinicians. There is an urgent need for a susceptible animal model of 2009 H1N1 infection that can be used to evaluate the pathogenesis and the therapeutic effect of corticosteroid treatment during infection.

**Methodology/Principal Findings:**

We intranasally inoculated two groups of C57BL/6 and BALB/c mice (using 4- or 6-to 8-week-old mice) to compare the pathogenesis of several different H1N1 strains in mice of different ages. Based on the results, a very susceptible 4-week-old C57BL/6 mouse model of Beijing 501 strain of 2009 H1N1 virus infection was established, showing significantly elevated lung edema and cytokine levels compared to controls. Using our established animal model, the cytokine production profile and lung histology were assessed at different times post-infection, revealing increased lung lesions in a time-dependent manner. In additional,the mice were also treated with dexamethasone, which significantly improved survival rate and lung lesions in infected mice compared to those in control mice. Our data showed that corticosteroid treatment ameliorated acute lung injury induced by the 2009 A/H1N1 virus in mice and suggested that corticosteroids are valid drugs for treating 2009 A/H1N1 infection.

**Conclusions/Significance:**

Using the established, very susceptible 2009 Pandemic Influenza A (H1N1) mouse model, our studies indicate that corticosteroids are a potential therapeutic remedy that may address the increasing concerns over future 2009 A/H1N1pandemics.

## Introduction

During the spring of 2009, a novel swine origin influenza A (H1N1) virus (S-OIV) emerged in Mexico and affected almost all countries in the world, bringing about the first influenza pandemic since 1968 [1], [2]. A considerable number of studies reported that there is a sharp difference in the age distribution of patients that exhibit disease following infections with pandemic 2009 influenza A (H1N1) and seasonal influenza viruses. The median age of hospitalized and fatal pandemic 2009 influenza A (H1N1) cases was approximately 16–27 years, which is younger than the age commonly associated with seasonal influenza; moreover, infants had the highest hospitalization rates [3], [4]. Different epidemiologic features in children and young people suggest that these age groups are more susceptible population of 2009 A/H1N1 infection. However, the pathogenesis associated with age during severe 2009 H1N1 infection has not yet been elucidated. Mouse models are important for characterizing pathogenesis and investigating therapies. We have piloted the use of a median lethal dose (LD50) of influenza A (H1N1) viruses in 4- and 6- to 8-week-old BALB/c and C57BL/6 mice in a previous study [5]. In this study, we wanted to assess the lung edema in mice at 4 weeks (weanling) and 6–8 weeks (young) of age to confirm the susceptibility of the mouse model and to shed light on the pathogenesis of 2009 A/H1N1 with respect to age. We also used the mouse model to screen for potential treatments for 2009 A/H1N1 infection in addition to the antiviral drug currently used.

It has been reported for decades that severe viral pneumonia can cause acute lung injury (ALI) and acute respiratory distress syndrome (ARDS) [6]. The 2009 A/H1N1 influenza pandemic also caused significant morbidity and mortality from acute respiratory failure [3], [7]. The major causes of ALI and ARDS following 2009 A/H1N1 influenza infection include the following: One major cause is attributed to cell death in the pulmonary parenchyma, as the lung epithelial cells may undergo apoptosis or autophagic cell death [8], [9], [10]. The second cause for the associated lung diseases is mediated by the exaggerated inflammation that occurs during infection. During virus infection, the host produces an immune response to eliminate the virions and reduce virus replication. In most cases, the viruses are cleared completely and the host gains complete recovery from infection. However, in some cases of over-responsiveness, the uncontrolled immune response plays a major pathological role in the disease process. Alveolar macrophages and domestic cells produce cytokines and chemokines, and these signals induce excessive recruitment of neutrophils to the lung with an over-expressed oxygen burst [11]. The exaggerated immune response and hypercytokinemia might be responsible for ARDS induced by 2009 A/H1N1 [12], [13]. Steroids have been prescribed for ARDS cases caused by different clinical diseases to decrease inflammation and inhibit the activity of the immune system, although the value of steroid treatment is currently disputed. Similar concerns remain with respect to the value of steroid treatment for H1N1 viral pneumonia-induced ALI/ARDS. For example, Dr Quispe-Laime et al. reported significant improvement in oxygenation and a relatively low mortality rate when using corticosteroids in patients with H1N1 influenza A virus-associated ALI [14]. Alternatively, Brun-Buisson [15] and Kim [16] maintain that corticosteroid therapy might harm patients by increasing viral loads and predisposing them to secondary infections. However, it is possible that clinicians tend to use steroids when patients get worse, which might conceal the possibility that steroid therapy would be beneficial [17]. Notably, Annane criticized that the retrospective cohort study used by the detractors is not a proper method to assess the efficacy of corticosteroids [18]. The use of corticosteroids as an adjunctive therapy for patients with severe H1N1 infection should be viewed as experimental and with extreme caution [19]. Salomon *et al.* have explored the potential of corticosteroid treatment against lethal H5N1 influenza infection in mice [20]. Although proinflammatory cytokines are also markedly elevated during H5N1 influenza virus infection, glucocorticoid treatment did not improve the outcome of infection in mice [20]. However, until now, mouse models have not been exploited to determine the efficiency of corticosteroid treatment during 2009 pandemic H1N1 infection. Thus, it still remains a conundrum as to whether clinicians should use corticosteroids in cases of severe influenza H1N1 infection. In this study, we used an established, susceptible mouse model to determine the efficacy of corticosteroid treatment for severe H1N1 infection-induced ALI and found that corticosteroid treatment ameliorated ALI.

## Materials and Methods

### Mice and Ethics Statement

Wild-type 4- and 6- to 8-week-old BALB/c and C57BL/6 mice were purchased from Vital River, Beijing, China. Mice were kept under specific-pathogen-free conditions and all mouse research were approved by the Institutional Animal Care and Use Committee of the Academy of Military Medical Sciences (ID: SYXK 2007-005). Operations including intranasal inoculation of live virus or AF and bronchoalveolar lavage (BAL) were performed under sodium pentobarbital anesthesia (60–80 mg/kg). The trachea was cannulated for BAL after anesthesia and tissues were handled gently. After BAL, the mice were euthanasized by cervical dislocation and then carefully observed and palpated to confirm that there was no respiration and heart beat. Mice showing >30% of body weight loss were considered to have reached the experimental end point that was approved by the Institutional Animal Care and Use Committee of the Academy of Military Medical Sciences and were humanely euthanized.

### Viruses and Reagents

The influenza viruses used in this study were A/Beijing/05/2006 seasonal H1N1 strain (seasonal H1N1), influenza A (H1N1) virus strain A/Beijing/501/2009 (BJ501) which is a pandemic H1N1 isolate, influenza A (H1N1) virus strain A/California/07/2009 (CA07), and influenza A (H1N1) virus strain A/PR/8/1934 (PR8). Live virus experiments using BJ501, CA07 and their mock-infected control, AF(P3), were performed in Biosafety Level 3 (P3) laboratory and live virus experiments using seasonal H1N1 and PR8 and their mock-infected control, AF(P2), were performed in Biosafety level 2 (P2) laboratory, under governmental and institutional guidelines. Viruses were propagated by inoculation into 9 to 10-day-old SPF embryonated eggs via the allantoic route. For BJ501, virus from earlier 3 generations was used. For UV inactivation, a 6-well plate containing 0.8 ml/well virus dilution of BJ501 was placed near the UV light (about 5 cm in distance) with the light on for 3 h in a biosafety cabinet.

### Viral Pathogenesis in Mice

#### Survival rate

4-week-old C57BL/6 mice were inoculated intranasally with 10^5.5^ TCID_50_ of virus or virus dilution. The survival in each group of ten mice was monitored daily for 10 days. Survival data were analyzed using Kaplan-Meier survival analysis.

#### Histological examination

After being anesthetized with pentobarbital sodium, 4-week-old C57BL/6 mice were inoculated intranasally with 10^5.5^ TCID_50_ of virus or virus dilution buffer and then sacrificed at various days post-infection (DPI). The lungs of each group of three mice were fixed in formalin and then embedded in paraffin. 4 µm sections were obtained and stained with hematoxylin-eosin.

#### Acute pulmonary edema (wet-to-dry ratio)

To assess the extent of acute pulmonary edema, the lung wet to dry weight ratios were calculated. 4-and 6-to 8-week-old BALB/c and C57BL/6 mice were used in this study. Six mice per group were anesthetized with pentobarbital sodium and intranasally inoculated with 10^5.5^ TCID_50_ virus or virus dilution buffer. At 5 days post-infection (DPI), the wet weight of the lungs from the mice was measured. The lungs were then heated to 68°C for 24 hours and the dry weight of the lungs was recorded, after which the wet to dry ratios were calculated.

#### Cytokine measurement

For cytokine measurement, the bronchoalveolar lavage fluid (BALF) of mice was collected after the mice were inoculated with virus or virus dilution buffer for 24 hours, and the BALF was processed with the IL-6 and TNF-α ELISA MAX™ Deluxe Sets (Biolegend, CA, USA).

### Dexamethasone Treatment of Viral Infection in Mice

4-week-old C57BL/6 mice were infected intranasally with 10^5.5^ TCID50 of BJ501 after being anaesthetized with pentobarbital sodium. Dexamethasone phosphate (1 mg/kg or 10 mg/kg) (Kingyork Company, Tianjin, China) or placebo were administered intraperitoneally 6 h before BJ501 infection and continued daily until the end of each experiment. Survival rate, histology, acute pulmonary edema and BALF cytokine levels were measured as described in the Viral Pathogenesis in Mice section.

### Statistical Analyses

All data are shown as the means ± S.E.M. Measurements at single time points were analyzed by ANOVA, and if they demonstrated significance, these points were further analyzed by a two-tailed t-test. Time courses were analyzed by repeated measurement (mixed model) ANOVA with Bonferroni post-t-tests. Survival data were analyzed using Kaplan-Meier survival analysis. All statistical tests were conducted using the GraphPad Prism 5 software. ***P***<0.05 indicates statistical significance.

## Results

### Four-week-old C57BL/6 Mice are the Most Sensitive to the 2009 A/Beijing 501 Strain

**Figure 1:**
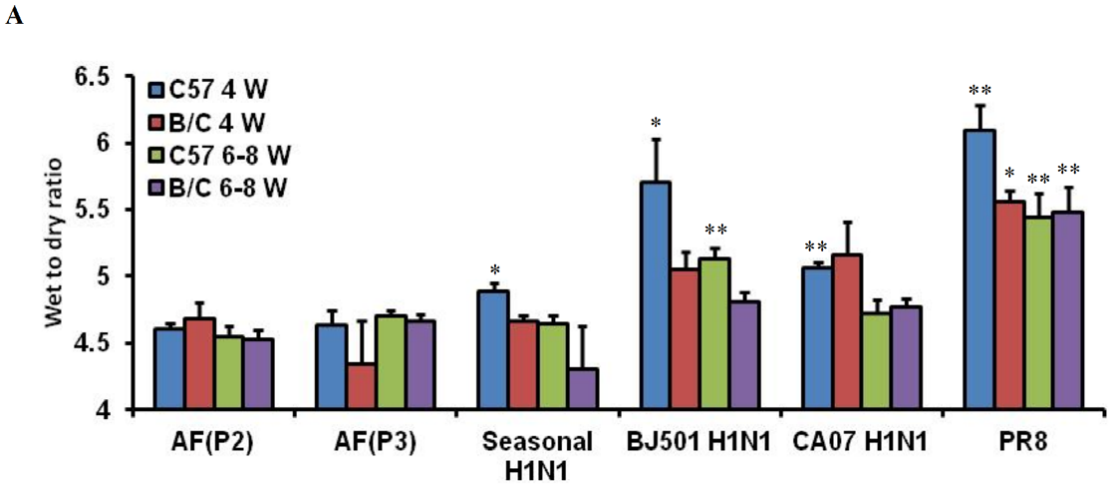
Establishment of a susceptible BJ501 infected mouse model. 4-week-old and 6- to 8-week-old C57BL/6 and BALB/c mice infected with 10^5.5^ TCID_50_ of Seasonal, BJ501, CA07, or PR8 viruses. Wet to dry ratios of lungs at 5 days post-infection with control, Seasonal, BJ501, CA07 or PR8 at 10^5.5^ TCID_50_ were measured. The BJ501 and PR8 induced ALI in mice. *P<0.05 and **P<0.01 compared with AF control. The values are means ± SEM from five mice.

Four H1N1 strains, including the seasonal H1N1, BJ501, CA07 and the commonly used mouse-adapted PR8 strain were used to infect 4- and 6- to 8-week-old C57BL/6 and BALB/c mice. The lung edema reflected by the wet/dry ratio of the lung tissue were investigated in our study. As a result, PR8 induced the most severe lung edema; BJ501 infection induced the next highest wet/dry ratios for most infection groups; whereas seasonal H1N1 infection did not induce any changes. CA07 infection had an intermediate virulence phenotype when compared to BJ501 and seasonal virus infection, causing relatively mild damage to the lung (Fig. 1).

We further compared the degree of lung injury in weanling and young mice. The wet/dry ratio changes for 4-week-old mice were significantly increased over strain-matched mice (Fig. 1). In particular, the C57BL/6 weanling mice exhibited the highest wet/dry ratios for both PR8 and BJ501 infections; these values were substantially higher than those exhibited by the BALB/c weanling mice (Fig. 1). Thus, we hypothesized that weanling C57BL/6 mice might be more sensitive to 2009 pandemic influenza infection compared to young C57BL/6 mice (Fig. 1). Therefore, weanling C57BL/6 mice were utilized for the influenza model represented in this study.

### Pathology and Cytokine Profiles in 4-week-old C57BL/6 Mice Infected with BJ501

**Figure 2:**
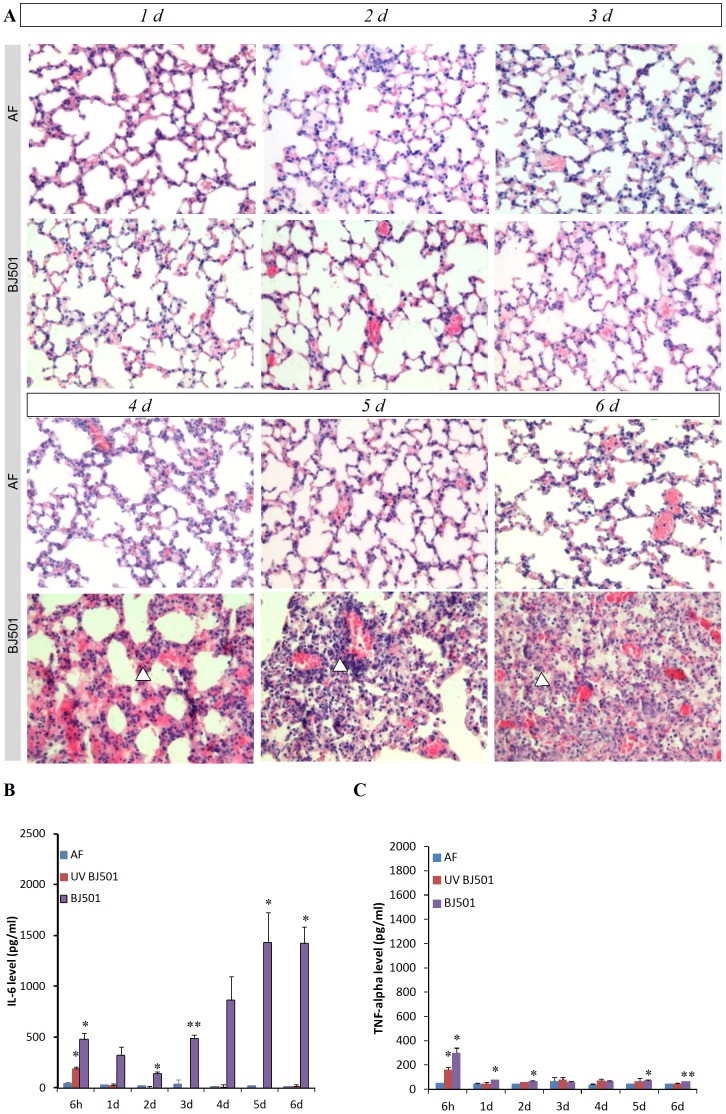
Effects of BJ501 exposure on lung injury and inflammatory responses in weanling C57BL/6 mice. Weanling C57BL/6 mice were infected with BJ501 at 10^5.5^ TCID_50_ or mock-infected. A, Lung histological findings of lung tissue in infected C57BL/6 mice. On day 4, 5, and 6 p.i., alveolar spaces were increasingly filled with alveolar epithelial debris, erythrocytes, and inflammatory cells (Δ). Magnification: ×200; B, C, Profiles of IL-6 and TNF-α in BALF samples. *P<0.05 and **P<0.01 compared with AF control. The values are the means ± SEM from five mice.

We infected weanling C57BL/6 mice with the BJ501 isolate to determine the pathological and cytokine changes that occur in the mice over multiple DPI. Pathological analysis of the mice showed progressive inflammatory reactions and lung lesions with bronchiolitis and alveolitis. Increased alveolar interstitial space, infiltrating cells, bronchitis with occasional necrosis of epithelial cells and alveolar hemorrhage can be easily recognized in the mice at 4 d, 5 d, 6 d after BJ501 infection (Fig. 2A lower panel); these results were in accordance with the elevated wet/dry ratio at 5 d (Fig. 1) and also with lung pathology in fatal human infection [21]. However, relative few changes could be observed in the first 3 days post-infection (Fig. 2A upper panel). The expression profiles of pro-inflammatory cytokines IL-6 and TNF-α in BALF after BJ501 infection were dramatically increased at 6 h. The concentrations of IL-6 and TNF-α were approximately 10- and 7-fold higher at 6 h, respectively, in the BJ501-infected group than in the control groups. IL-6 levels fluctuated but remained significantly elevated during the observation period and reached peak levels at day 6 post-infection, which was the time point when mice began to succumb to infection; these findings were consistent with the clinical symptoms observed. TNF-α levels decreased after 24 h and did not increase greatly in subsequent days, suggesting a function in the launching of the deregulated inflammation. Weanling C57BL/6 mice were also infected with the UV inactivated BJ501 virus to determine whether the inflammatory effect is due to infection or just in response to inoculation with high concentrations of foreign protein. There is a temporal response to UV inactivated virus at 6 h, but this temporal response rapidly recovered after 1 d of inoculation (Fig. 2B, 2C).

In summary, these studies demonstrated that BJ501 virus strain induced high levels of virulence, causing ALI and a high degree of hypercytokinemia in infected weanling C57BL/6 mice. Thus, we hypothesized that in the cases of 2009 pandemic influenza infection, the host produced an over-exaggerated immune response and that the cytokine storm might play a critical pathological role in the disease.

### Dexamethasone Treatment Ameliorates ALI Induced by BJ501 Infection

**Figure 3:**
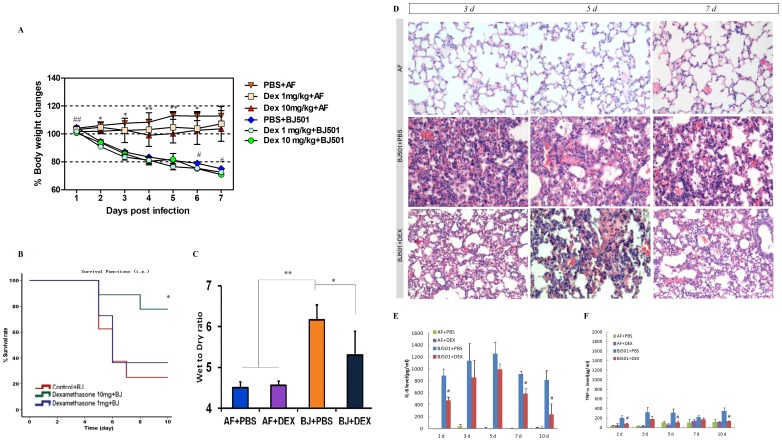
Administration of Dexamethasone phosphate ameliorated ALI in mice. **A,** Changes in body weight. The values are Means ± SEM from five mice. *P<0.05, **P<0.01, AF inoculated mice treated with 10 mg per kg per day dexamethasone compared with those treated with PBS. #*P*<0.05, ##*P*<0.01, BJ501 inoculated mice treated with 10 mg per kg per day dexamethasone compared with those treated with PBS. **B,** Mortality rates of C57BL/6 mice intraperitoneally injected with control or 1 mg, 10 mg per kg per day dexamethasone phosphate for 10 successive days. Ten mice per group were used in these experiments. *P<0.05 compared with placebo control. **C,** Wet to dry ratios of lungs at 5 days post-infection with BJ501 at 10^5.5^ TCID_50_ with intraperitoneal administration of control or 10 mg per kg per day dexamethasone phosphate for 5 successive days. The values are means ± SEM from six mice. *P<0.05 and **P<0.01. **D,** Representative pathological images of the lungs of mice infected with BJ501 at 10^5.5^ TCID_50_ and intraperitoneally treated with control or 10 mg per kg per day dexamethasone phosphate for 3, 5, or 7 successive days. Magnification: ×200. **E,** **F,** Profiles of IL-6 and TNF-α in BALF samples of mice infected with BJ501 at 10^5.5^ TCID_50_ and intraperitoneally treated with control or 10 mg per kg per day dexamethasone phosphate for 1, 3, 5, 7,or 10 successive days. The values are means±SEM from six mice in BJ501 groups and three mice in AF groups. #*P*<0.05, BJ501 treated with 10 mg per kg per day dexamethasone compared with BJ501 treated with PBS.

Corticosteroid administration is among the most powerful treatments used to curb inflammation. Considering the unusual high cytokine levels assessed in the above referenced data and the clinical hypercytokinemia reported [12], [13], we hypothesized that corticosteroid treatment might serve to control the pneumonia induced in the mouse model. Moreover, during the 2009 H1N1 pandemic, corticosteroid treatment was commonly prescribed besides antiviral drugs such as oseltamivir [14]. However, controversial views have been reported with respect to the efficacy of corticosteroids in treating ARDS patients infected with 2009 pandemic H1N1 virus [15], [16], [17], [18], [22], [23]. Using our mouse model of the 2009 pandemic BJ501 infection, we confirmed the efficacy of dexamethasone for the treatment of 2009 pandemic infections by assessing the survival rate, wet/dry ratio, pathology and the BALF cytokine levels. Mouse body weight loss in the PBS and dexamethasone treatment group after BJ501 infection were 26% and 29.2%, respectively. A daily injection of dexamethasone also caused body weight loss due to side effects (Fig. 3A). The survival rate of the mice was substantially improved when they were treated with dexamethasone (10 mg/kg) daily for 10 continual days (Fig. 3B). Furthermore, a dose-dependent relationship can also be observed in the survival rate improvement following dexamethasone treatment (Fig. 3B). The wet/dry ratio and lung pathology, reflecting ALI, were also improved, accompanied with reduced BALF IL-6 and TNF-α levels, when mice were continually administered dexamethasone (10 mg/kg per day) until euthanization (Fig. 3C, 3D, 3E). These results indicated that inflammation played an important role in BJ501-induced ALI in mice and that the effects could be ameliorated by suppressing pro-inflammatory cytokine levels with corticosteroid treatment *in vivo*.

## Discussion

The 2009 influenza A (2009 H1N1) pandemic caused illness with severe complications, including fatality, worldwide. Many clinical reports have indicated that, in comparison with seasonal influenza, pandemic 2009 influenza A (H1N1) disproportionately affects younger individuals and that infants and children infected with influenza are more likely to develop complications compared to adults [3]. However, the pathogenesis of 2009 H1N1 infections in younger individuals remains unclear. Because 4-week-old mice are more similar in age to infants and children than 6- to 8-week-old mice, it is reasonable to anticipate that 4-week-old mice are more sensitive to 2009 A/H1N1 virus. In our pervious study, we have found that infection with the BJ501 isolate had a LD50 at a virus titer of log10 1.9 in 4-week-old C57BL/6 mice, which was substantially less than that in 6-to 8-week-old C57BL/6 mice [5]. In the present study, 4-week-old mice exhibited significantly higher wet/dry ratios compared to strain-matched 6-to 8-week-old mice in most cases. Our data supported that the infection was more severe in 4-week-old mice than in 6-to 8-week-old mice. It is known that H1N1 infection might induce an over-exaggerated immune response and that the weanlings have relatively immature immune system. However, the weanlings showed more severe impairment in lung function. Perhaps other factors are involved in lung injury, such as immature lung epithelium or surfactant protection ability, making the lungs of the weanlings more susceptible to H1N1 infection. On the other hand, infection with the 2009 H1N1 strain may induce an aberrant pulmonary immune response specifically in the weanlings.

Mice infected with the typical 2009 H1N1 virus were reported to exhibit mild lung lesions and inflammation [24], [25], similar to the CA07 strain that showed limited virulence in our study (Fig. 1). SD-09, a virulent 2009 H1N1 variant isolated from swine, caused severe lung damage and induced ARDS in mice [26]. We isolated the virulent pandemic H1N1 isolate, BJ501, from a patient with severe H1N1 infection in Beijing, China, during the 2009 pandemic. The strain was found to cause more severe respiratory disease than the CA07 strain in mice, showing severe edema (Fig. 1). Consequently, the weanling C57BL/6 mouse model of BJ501 infection was established to study the associated lung lesions and the therapeutic efficacy of corticosteroid treatment.

Mice that were infected intranasally with 10^5.5^ TCID_50_ of BJ501 virus showed obvious respiratory symptoms, most importantly causing lethal disease. The lungs of virally infected mice were highly edematous, which was also demonstrated by a dramatically increased lung wet/dry weight ratio. Pathological changes rendered a progressive pattern with diffuse alveolar damage, hemorrhage, interstitial edema and extensive inflammatory accumulation from 4 to 6 DPI (Fig. 2). It is reported that the lung is a major source for cytokine storms in patients with an inflammatory lung process [27]. Alveolar macrophages up regulate pro-inflammatory cytokines that may further damage alveolar pneumocytes and may play a critical role in disease pathogenesis [11]. In particular, IL-6 and TNF-α are the primary contributors to hypercytokinemia and were significantly increased following 2009 H1N1 virus infection [21], [26], [28], [29]. As a sign of alveolar macrophage activation, the concentrations of secreted IL-6 and TNF-α in the BALF were significantly up regulated, in the weanling C57BL/6 groups infected with BJ501. These results suggested that BJ501 also induced the production of pro-inflammatory cytokines that are reflected in the human clinical disease, which may cause a cytokine storm that leads to ALI in mice.

Corticosteroids, the immune-modulators, have strong anti-inflammatory activities and are widely prescribed for many immune and inflammatory diseases [30]. There is also a strong biological rationale to sustain the use of corticosteroids for H1N1 influenza-induced pneumonia and ARDS because the 2009 H1N1-induced ALI was characterized by uncontrolled excessive lung and systemic inflammation that induced lung damage rather than uncontrolled viral infection [18], [21], [31]. Corticosteroids were broadly used in patients with severe pneumonia and ARDS during the 2009 H1N1 pandemic; nevertheless, there have been conflicting reports on the effects of the treatment [14], [15], [16], [17], [18], [19], [32], [33], [34]. It is recommended by WHO that high doses of systemic corticosteroids should not be used for severely ill H1N1-infected patients, because of the possibilities of opportunistic infection, viral shedding and prolonged viral replication [1]. However, evidence from a severe patient that was resistant to oseltamivir but significantly improved after corticosteroid infusion showed that corticosteroid did not cause a harmful increase in viral load during severe H1N1 virus infection, supporting the use of corticosteroid in patients affected by severe H1N1 [34]. Moreover, the 2011 May 1 issue of *American Journal of Respiratory and Critical Care Medicine* also presented the arguments, including both pros and cons, on the concept of corticosteroid treatment for ALI associated with H1N1 viral pneumonia within different groups [15], [16], [17], [18]. None of the present clinical studies reporting on treatment strategies for patients with H1N1 from the 2009 pandemic was randomized, double-blind clinical trials, which is the only proper method for assessing the efficacy and safety of any drug for any disease; they were instead observational studies, and many confounding issues remain to be addressed [15], [16], [18]. There is also no experimental evidence in mice reported in the literature on the use of corticosteroid in H1N1 viral pneumonia, thus highlighting the importance of our study in establishing a mouse model to evaluate corticosteroid effects. Our results using the susceptible weanling C57BL/6 model of BJ501 infection revealed positive consequences for corticosteroid treatment in H1N1 viral pneumonia. The results of survival rate, pathology and lung edema all pointed to a better fate for the corticosteroid treatment groups, supporting the use of corticosteroid to treat viral pneumonia. However, a randomized, double-blind clinical trial is still in urgent need to provide sufficient evidence to persuade clinicians to administer steroids to patients with ARDS from viral pneumonia.

In conclusion, weanling C57BL/6 mice infected with the BJ501 isolate proved to be an ideal animal model for the severe cases of 2009 pandemic influenza infection in humans. Our experimental results from the mouse model of 2009 A/H1N1 infection suggested that corticosteroid treatment could be useful in patients infected with the 2009 pandemic influenza virus and help us to be better prepared for the next influenza pandemic.
